# Seismic shifts in the geochemical and microbial composition of a Yellowstone aquifer

**DOI:** 10.1093/pnasnexus/pgaf344

**Published:** 2025-11-25

**Authors:** Eric S Boyd, Daniel R Colman, Ana Menchaca, Rachel L Spietz, Anna Shoemaker, Carol Finn, David Mencin, Eva Andrade-Barahona, Alysia Cox, Thomas Kieft, Susan Bilek, Jefferson Hungerford, Tullis C Onstott

**Affiliations:** Department of Microbiology and Cell Biology, Montana State University, 109 Lewis Hall, Bozeman, MT 59717, USA; Department of Microbiology and Cell Biology, Montana State University, 109 Lewis Hall, Bozeman, MT 59717, USA; Department of Microbiology and Cell Biology, Montana State University, 109 Lewis Hall, Bozeman, MT 59717, USA; Department of Microbiology and Cell Biology, Montana State University, 109 Lewis Hall, Bozeman, MT 59717, USA; Department of Microbiology and Cell Biology, Montana State University, 109 Lewis Hall, Bozeman, MT 59717, USA; Geology, Geophysics, and Geochemistry Center, United States Geological Survey, 6th Avenue and Kipling Street, Denver, CO 80225, USA; Department of Geosciences, University of Colorado, 2200 Colorado Avenue, Boulder, CO 80309, USA; Department of Chemistry and Geochemistry, Montana Technological University, 1300 West Park Street, Butte, MT 59701, USA; Department of Chemistry and Geochemistry, Montana Technological University, 1300 West Park Street, Butte, MT 59701, USA; Department of Biology, New Mexico Tech, 801 Leroy Place, Socorro, NM 87801, USA; Department of Earth and Environmental Sciences, New Mexico Tech, 801 Leroy Place, Socorro, NM 87801, USA; National Park Service, Yellowstone Center for Resources, Yellowstone National Park, PO Box 168, WY, USA; Department of Geosciences, Princeton University, 320 Guyot Hall, Princeton, NJ 08544, USA

**Keywords:** earthquake, volcano, autotroph, hydrogen, organic carbon

## Abstract

Seismic energy, like that released by earthquakes, can fracture rock and thereby alter subsurface fluid flow paths, release substrates from inclusions, and expose fresh mineral surfaces capable of reacting with water. However, it is unclear how such seismic-induced changes influence microbial communities. Volcanically active areas experience frequent seismic activity and thus represent ideal locations to examine the influence of seismic-induced geochemical change on subsurface microbial communities. Here, we demonstrate that energy released in an earthquake swarm in 2021 correlated with extensive temporal change in the geochemical and microbial composition of aquifer fluids sampled from ∼100 m depth in a borehole in Yellowstone National Park. Increased energy absorbed at the borehole over time was correlated with increased concentrations of hydrogen, dissolved organic carbon, and sulfide and was associated with depletion of δ^13^C in dissolved organic carbon, increased concentrations of cells, and increased abundances of chemolithotrophic, putative hydrogen-oxidizing *Dethiobacteraceae* and *Desulfotomaculum* bacteria. Dissipation of the earthquake swarm was associated with decreased concentrations of hydrogen, sulfide, and cells. These results suggest the subsurface biosphere dynamically responds to seismic-induced geochemical change at the level of activity and growth. Laboratory mechanical comminution of rhyolite, the primary bedrock in Yellowstone, released organic carbon and hydrogen and generated hydrogen when exposed to water. This indicates the presence of a large subsurface reservoir of organic carbon and hydrogen that can be released or generated by seismic induced bedrock fracturing. Taken together, these data indicate seismic-induced generation of chemical disequilibria can support the persistence of complex subsurface microbiomes.

Significance The subsurface hosts a substantial fraction of Earth's biomass and biodiversity. Unlike surface ecosystems that are supported by light energy, subsurface ecosystems are fueled by chemical energy released by dissipation of disequilibria in chemical reactions. However, it is unclear how chemical disequilibria is maintained in the subsurface over geological time periods to support abundant and diverse microbial communities. Here, we show that energy released by earthquakes in Yellowstone generates chemical disequilibria that microbial inhabitants respond to at the level of activity and growth. These results provide new insight into how microbial life is maintained in Earth's subsurface, both today and in its distant past, and suggest similar geologic processes could support subsurface life on seismically active planets, like Mars.

## Introduction

The subsurface hosts up to 30% of Earth's biomass (1–3) and its biosphere has been suggested to extend to depths of >5 km if clement conditions for microbial life are maintained (4). Subsurface microbial communities are fueled by disequilibria in chemical reactions involving hydrogen (H_2_), carbon dioxide (CO_2_), and a variety of sulfur and iron chemicals, among others (4–8). Disequilibria among such chemicals are maintained largely by water-rock interaction including the processes of radiolysis (e.g. (9)), cataclasis (e.g. (10–12)), and serpentinization (e.g. (13, 14)). Reactants on mineral surfaces responsible for maintaining chemical disequilibria, however, are consumed during water–rock reactions (11). This raises the key questions of how, when, and where such chemicals are generated/released in the subsurface and how disequilibrium is maintained over geologic time periods to support isolated communities founded on chemical (mineral) energy.

Kinetic energy, like that released by earthquakes can fracture rock thereby altering fluid flow paths, releasing substrates in inclusions or pore spaces, and/or exposing fresh mineral surfaces capable of reacting with water (15–17). Numerous studies have documented broad changes in the geochemistry of aquifer fluids following earthquakes (18–23). Of particular significance to microorganisms are geochemical changes induced by the mechanical shearing of silicate minerals, that once exposed to water, have been shown to initiate a series of radical reactions that can produce H_2_, oxygen, and sulfate (SO_4_^2−^), among other redox-active compounds (10–12, 24–28). Increased water–rock interaction made possible by natural, seismic-induced bedrock fracturing may explain the increased prevalence of H_2_ and other geogases such as CH_4_ in fluids from active fault zones (11, 28–31). Similarly, bedrock fracturing due to mining activities was associated with increased concentrations of H_2_ and CH_4_ in a 3.54 km deep borehole (32). Given that the borehole was dry, it is likely that alteration of fluid flow paths by induced bedrock fracturing increased the connectivity of isolated fluids, allowing for release of H_2_ and other geogases that have accumulated in the crust (33–36).

While the effect of seismic activity on the geochemistry of fluids has been widely studied, far less is known of the influence of seismic activity on microbial communities. One study examined the taxonomic composition of microbial communities in aquifers four months after a magnitude (M) 5.8 seismic event (37). While the composition of the communities changed slightly during the sampling period, it is not clear if this was due to geochemical change associated with the seismic event itself. Other studies have speculated about the influence of seismic H_2_ on subsurface microbial ecosystems based on ex situ geochemical or cultivation assays (26, 38, 39) or in situ activity assays (31), the latter of which identified an increased abundance of H_2_-dependent acetogens in sediments near faults 1 year after the M 9.1 Tōhoku-oki earthquake off the coast of Japan. As such, the influence of seismic activity on subsurface microbial communities is unknown and the critical question of whether seismic induced changes in geochemistry (e.g. generation or release of H_2_) impact microbial activity and biomass production in subsurface ecosystems remains unanswered. This knowledge gap can be largely attributed to logistical challenges in coordinating temporal sampling before, during, and after unpredictable and often rare seismic events.

**Fig. 1.**
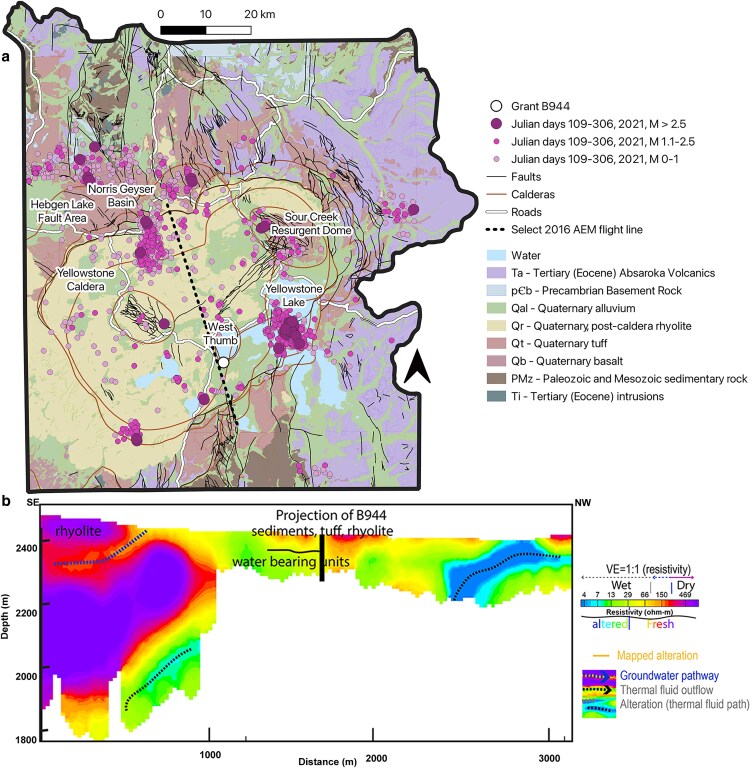
a) Location of the Grant (B944) borehole in Yellowstone National Park (YNP). Magenta dots represent seismic events across Julian days 109–306, 2021, with the size indicative of the magnitude (M) of the event. Quaternary fault lines are shown in black, caldera boundaries are shown in brown, and a single aerial electromagnetic (AEM) flight line that is 1200 m west of B944 is shown with a black dashed line (40). b) Cross-sections from 1D electrical resistivity models from inverted AEM data collected along the selected flight profile. Groundwater and thermal fluids containing appreciable total dissolved solids significantly reduce resistivities of porous volcanic rocks and can be differentiated by their resistivity signatures (40). Mapped alternation and resistivity signatures of groundwater pathways, thermal fluid pathways, and alteration are depicted.

Earthquakes tend to be concentrated near plate margins and volcanically active areas (11, 28) and are frequent in Yellowstone Plateau Volcanic Field (YPVC), United States (Fig. 1a), where ∼1,000 to 3,000 events occur per year (15). These seismic events are dominated by earthquake swarms (41), defined as the spatial and temporal clustering of earthquakes, which have most recently occurred in a north–south trending belt between the Sour Creek resurgent dome and Yellowstone Lake (42) and near the northwest rim of the caldera, proximal to Norris Geyser Basin (41). Seven boreholes were drilled in the YPVF to monitor the volcano and seismic activity (43), one of which is located on the western shore of Yellowstone Lake (Grant B944) (44). Numerous faults and fractures, both mapped and buried, as well as lateral boundaries between flows and in glacial deposits, provide permeable pathways for fluid migration at the B944 well (40, 43). More specifically, the near surface of B944 is complex and comprises glacial and tuffaceous sediments (surface to ∼13.7 m) that overly thicker rhyolitic lava flows that comprise welded and unwelded rhyolitic tuff (43). Resistivity data suggest the presence of near surface water bearing units that could facilitate lateral fluid flow near B944 (Fig. 1b) (40). Here, temporal relationships between radiated seismic energy from local earthquakes and changes in the chemistry and microbial communities across 6 months are examined in an aquifer hosted in rhyolitic bedrock that is intersected by B944. Further, we characterize altered and unaltered hand samples of rhyolite and determine the amount of H_2_, CH_4_, and organic carbon released following dry mechanical comminution (release of substrates from inclusions, pore spaces) and exposure to water (generation of substrates via water–rock interaction). The hypothesis guiding this work was that chemical changes to the aquifer, either through mineral resurfacing and subsequent water-rock interaction, alteration of fluid flow paths, or release from fluid inclusions or pore spaces in rock, would stimulate microbial activity and growth.

## Materials and methods

### Generation of geologic map

Map layers were obtained from the Wyoming State Geological Survey and were modified from the Digital Geologic-GIS Map of the Yellowstone National Park and Vicinity, Wyoming, Montana, and Idaho adapted from US Geological Survey maps by Christiansen, Blank, Prostka, Smedes, Pierce, the US Geological Survey, Elliot, Nelson, Wahl, Witkind, Love, and others (1956 to 2007), and a Montana Bureau of Mines and Geology map by Berg, Lonn, and Locke (1999) in the software QGIS 3.30.1. These layers are available from the National Park Service Geologic Resources Inventory program (Lakewood, CO; https://irma.nps.gov/DataStore/Reference/Profile/1044842).

### Site description and sample collection

B944 (44.389669, −110.543722; Elevation, 2365 m) is located south of the West Thumb of Yellowstone Lake and permitted (YELL-08154) access is via a National Park Service (NPS) road. B944 was drilled between 2008 July 16 and July 26 to a depth of ∼157 m. Sample collections were performed in 2021 on May 27 (Julian Calendar date 147), June 23 (174), August 25 (237), October 6 (279), and November 3 (307); sampling in July was attempted but not possible due to mechanical failure of the bladder pump. Julian Calendar dates are used from herein to facilitate continuous time reporting in 2021. Samples were collected from a depth of 99.1 m using a model 407 bladder pump (Solinst, Georgetown, Canada) equipped with a polytetrafluoroethylene bladder. The bladder pump was equipped with a high density polyethylene (HDPE) sample line, a HDPE drive line, and a stainless steel recovery cable. The sample line was rinsed in the lab with ∼100 L of distilled water before and after each use. Fluids were collected by displacement using compressed air with sample collection and bladder refilling times optimized for sample depth (per manufacturer guidelines). Compression and inflation of the bladder was automatically controlled during sampling using a model 464 Mk3 electronic control unit (Solinst) and a high pressure compressor. A model 3001 Levelogger 5 (Solinst) was used to measure temperature at the depth of sampling.

The bladder pump sample line (at the surface) was equipped with a Luer-lok fitting and a three-way stopcock to enable sample collection without atmospheric exposure. Prior to sample collection, a minimum of 2 L of water was pumped through the bladder and sample line (total volume for both is 1.4 L); samples were only collected once the conductivity (YSI EC300, Yellow Springs, OH) of sampled waters stabilized. Planktonic biomass samples for molecular analyses were collected using 0.22-µm polyethersulfone (PES) Sterivex filters (Millipore; Burlington, MA); filtrate was collected for geochemical analyses (described below). Depending on the sampling date and amount of biomass, between 50 and 100 mL of water could be filtered through each Sterivex filter before back pressure resulted in bladder failure. A minimum of three filters of biomass were collected at each sample date from each well. Following sample collection, filters were purged with 160-mL of ultra-high purity N_2_ using a sterile syringe, were placed in sterile 50-mL Falcon tubes, and were frozen on site using dry ice. Filters remained frozen (−80 °C) until used for molecular analyses.

Filtered (0.22 µm Sterivex) water samples for inductively coupled plasma-mass spectrometry (ICP-MS) for trace metal determination, ICP-optical emission spectrometry (OES) for major cation determination, and ion chromatography (IC) for anion determination were collected in low density polyethylene (LDPE) bottles. Prior to sample collection, bottles were washed with trace metal grade nitric acid and were rinsed with MilliQ (MQ) water (three times). Following collection, samples were immediately acidified (1% vol/vol) with trace metal grade nitric acid. Samples for anion analyses via ion chromatography were collected as above but were not acidified. Samples were sent to the Analytical Laboratory at the Montana Bureau of Mines and Geology for ICP-MS (Thermo Scientific iCAP Q; Waltham, MA), ICP-OES (Thermo Scientific iCAP 6000), and IC (Metrohm Compact IC Plus; Herisau, Switzerland) analyses, as described previously (45). Samples for dissolved organic carbon (DOC) quantification and isotopic analyses were filtered using 0.22-µm Supor filters and were collected in pre-ashed, brown glass bottles. DOC concentrations were initially determined via a Shimadzu TOC-V_CSH_ analyzer (Kyoto, Japan). Secondarily, DOC concentrations and their carbon isotopic compositions were determined using a Picarro G2131-I and Gas Concentration Analyzer (Santa Clara, CA) coupled to an Aurora 1030W Total Carbon Analyzer (YSI, Yellow Springs, OH). Samples of MQ water were filtered in the field to assess (via Shimadzu) background DOC associated with filtering. In the lab, samples of MQ water were pumped using the PTFE bladder and 500' HDPE sample line, were filtered as above, and analyzed (via Shimadzu) to assess background DOC levels associated with the bladder and sample lines. DOC concentrations from these controls never exceeded 0.4 mg L^−1^. Samples for δ^2^H and δ^16^O analysis of water (Picarro model L2130-I) were collected in autoclave-sterilized LDPE bottles with no headspace and were not acidified. All filtered waters were immediately placed on ice in the field and stored at 4 °C prior to analysis. Dissolved (0.22 µm-filtered) and total (unfiltered) sulfide and Fe(II) concentrations were measured on-site using reagent-based colorimetric tests via the methylene blue (Hach method 8131) and ferrozine assays (Hach method 8147) and a model DR890 portable spectrophotometer (Hach Company, Loveland, CO).

Ten milliliter samples of unfiltered water were collected into 15-mL Falcon tubes containing 0.22 µm-filtered formalin (2% final concentration), immediately placed on ice in the field, and stored at 4 °C prior to cell enumeration. Within 3 days of sample collection, fixed cells were stained by adding 4',6-diamidino-2-phenylindole (DAPI) to a final concentration of 2 µg/mL followed by incubation at room temperature (∼21 °C) for 15 min. Samples were then treated with detergent [100 mM ethylenediaminetetraacetic acid (EDTA), 100 mM sodium pyrophosphate, 1% (v/v) Tween 80] to disaggregate cells as previously described (46). Disaggregated and stained cells were filtered onto black 0.22 µm polycarbonate filters (MilliporeSigma, Burlington, MA) and were enumerated using an Evos fluorescence microscope (ThermoScientific, Waltham, MA).

Unfiltered waters were also collected for measuring dissolved gases using a modified bubble strip method, as described previously (47). Briefly, 100 mL of water was collected directly from the Luer-lok fitting on the sample line using a 160-mL syringe equipped with a 3-way stopcock. A 10-mL volume of ultra-high purity (UHP) N_2_ was then injected into the syringe and the syringe contents were vigorously shaken for 10 to 15 min to promote equilibration of dissolved gases into the headspace bubble. The gas bubble was then collected via displacement into 24-mL serum bottles containing saturated sodium chloride and were stored at room temperature (47). At the lab, 10 mL of the gas bubble were injected into a model SRI 8610C gas chromatograph (SRI instruments, Torrance, CA) and gases were measured as previously described (47). Helium was used as a carrier gas for all analyses. Gas concentrations were calculated using certified standards (American Gas Group, Toledo, OH) and were converted to dissolved aqueous phase concentrations using Henry's law. All values are presented as the average of the mean of three replicates. Extensive gas exsolution due to depressurization during sample collection prevented accurate pH measurements of waters discharged at the surface. Thus, pH paper housed in a stainless-steel mesh cylinder was lowered into the well to qualitatively determine pH at the top of the water column. Select geochemical data are reported in Table 1, and all geochemical data is presented in SI Appendix Table S1.

**Table 1.** Select geochemical and microbial data collected on fluids sampled from the Grant B944 borehole (Lat: 44.389669; Long: −110.543722; Elev: 2365 m) on the specified dates. Full geochemical and microbial datasets are provided in Table S1.

	Grant (B944) Coordinated Universal Time (UTC) and Julian Calendar Date				
	2021 May 27	2021 June 23	2021 August 25	2021 October 6	2021 November 3
Analyte	147	174	237	279	307
Temp. (°C)	35	35	35	35	35
pH	7.5	7.5	7.5	7.5	7.5
Cond. (µS cm^−1^)	355.9	306.4	326.9	230.6	230.0
HS^−^ (µM; unfilt)	10.3 (0.8)	44.8 (5.5)	57.8 (1.6)	62.5 (4.1)	43.2 (0.9)
HS^−^ (µm; filt)	3.1 (0.0)	26.6 (2.7)	44.3 (2.4)	49.0 (1.8)	34.4 (3.1)
Fe(II) (µM; unfilt)	2.9 (0.4)	4.1 (0.5)	4.0 (0.6)	1.7 (0.1)	0.8 (0.2)
Fe(II) (µM; filt)	0.3 (0.0)	0.4 (0.0)	0.6 (0.1)	0.2 (0.1)	BD
H_2_ (µM)	30.6 (1.5)	26.7 (0.2)	44.0 (0.2)	41.6 (0.7)	23.4 (1.3)
CO_2_ (nM)	BD	BD	BD	BD	BD
CH_4_ (nM)	BD	BD	BD	BD	BD
δ^18^O H_2_O VSMOW (‰)	−15.2	−15.4	−16.0	−15.9	−16.3
δ^2^H H_2_O VSMOW (‰)	−124.8	−126.7	−128.6	−127.8	−127.3
DOC (mg L^−1^)	3.46 (0.32)	4.32 (0.88)	NA	7.90 (0.40)	NA
δ^13^C DOC PDB (‰)	−14.0 (0.5)	−14.2 (0.9)	NA	−17.5 (0.4)	NA
Cl^−^ (µM)	271	293	313	322	347
SO_4_^2−^ (µM)	65	123	27	51	27
Cells mL^−1^ (avg)	1.28 ± 0.31 × 10^6^	3.11 ± 0.44 × 10^6^	8.36 ± 0.31 × 10^6^	8.83 ± 0.21 × 10^6^	2.23 ± 0.10 × 10^6^

The standard deviation of triplicate measurements is provided in parentheses, where available.

### DNA extraction and metagenomic sequencing

Filters were removed from Sterivex cartridges using a sterile autoclaved saw, as previously described (48). DNA was extracted from triplicate filters containing biomass using the FastDNA Spin Kit for Soil (MP Biomedicals, Santa Ana, CA) and was quantified with the Quant-iT DNA HS assay and a Qubit fluorometer (Invitrogen, Carlsbad, CA). Sterile filters were also processed and subjected to extraction to assess potential contamination associated with these steps; quantifiable DNA was never measured in blank extractions. Equal volumes of each triplicate extract were then combined and frozen at −20 °C for use in metagenomic sequencing. Illumina library preparation and paired-end NovaSeq6000 sequencing (2 × 150 bp) were conducted at the University of Wisconsin Sequencing Center.

Reads were trimmed with the TrimGalore v.0.6.0 program to cleave sequencing adapters, followed by down-sampling with bbnorm (https://github.com/BioInfoTools/BBMap; v.38.87) to account for sequence redundancy and improve assemblies, as previously described (45). Trimmed and down-sampled sequences were assembled individually using the metaSPAdes v.3.14.0 assembler and with default parameters. Assembly statistics are reported in SI Appendix Table S2. Assembled contigs were binned into metagenome-assembled genomes (MAGs) using the MetaWRAP v.1.3.2 pipeline based on read depth and tetranucleotide frequency and independent binning with the metaBAT v.2, MaxBin v.2, and CONCOCT v.1.1.0 algorithms, and by specifying a length (l) of −2500. Optimal MAG bin datasets were identified using the bin refinement module of MetaWRAP. Only MAGs that exhibited >50% estimated completeness and <10% contamination (consistent with moderate to high quality genomes (49)) were retained for further analyses.

MAGs were taxonomically classified using the GTDB-Tk v.1.3.0 classifier (50) and the bac120 and arc122 datasets for bacterial and archaeal classification, respectively. Taxonomic designations are provided to the lowest taxonomic rank that was formally recognized at the time of the writing of this manuscript. The relative abundance of MAGs was estimated based on mapping of quality-filtered reads to those MAGs using the BWA mapping tool (v.0.7.17). Relative abundances are reported as the percentage of reads mapped to MAGs. To evaluate whether the MAG-based dataset accurately represented community taxonomic composition, MAG taxonomic classifications and their associated relative abundances were compared against estimates from assembly-free read-based taxonomic analyses, as described previously (45). MAG contigs and the metagenomic reads have been deposited in the National Center for Biotechnological Information (NCBI) whole genome sequence (WGS) database under bioproject accession PRJNA1164288. MAG taxonomy, estimated completeness, contamination, and abundance are reported in Tables S3 and S4.

MAGs from each depth were collapsed into operational taxonomic units (OTUs) specifying a threshold of >95% average nucleotide identity (ANI), using the fastANI program (v.1.32) (51). A representative MAG from each OTU was selected for downstream analyses. MAGs were first selected to maximize estimated genome completion and secondly to minimize estimated contamination. MAG gene predictions were made with PROKKA v 1.11 (52) and resultant protein annotated files were then subjected to metabolic reconstruction first via the Hidden Markov Model (HMM) searches of key functional genes, as implemented in the METABOLIC software program (v.4.0) (53). Whole metagenome assemblies were also subjected to protein annotation using PROKKA using the “metagenome” parameter flag and minimum contig lengths of 1,000 bp. Refinement of metabolic annotations was conducted, as previously described (45). [NiFe]- and [FeFe]-hydrogenases were identified in the unassembled metagenome assemblies and MAG bins by BLAST searches of query sequences, as described elsewhere (54), alongside the HMM-based identifications within METABOLIC (Table S5). Briefly, candidate homologs were identified based on >30% amino acid identity and >60% query coverage to the query sequences. The presence of conserved cysteinyl motifs and affiliation with known groups of hydrogenases were evaluated based on comparison to the HydDB database (55), with manual verification with our in house hydrogenase database (54). The BLAST-based and HMM-based identifications were combined to represent the total set of hydrogenases.

### Mineral coupons and 16S rRNA gene sequencing

Hand samples of altered and unaltered Lava Creek Tuff (rhyolite) were kindly provided by Dr. Madison Myers from a personal NPS-permitted collection. Rhyolite is the predominant bedrock type at >13 m depth in B944 (43). The unaltered rhyolite sample was coated in 70% ethanol and then rinsed with filter-sterilized MQ water in a UV-treated laminar flow hood and was then crushed with an ethanol (70%)-treated hammer. A model LC-8 jaw crusher equipped with chromium- and carbon-hardened stainless-steel plates (Gilson, Lewis Center, OH) was then used to crush the fragments into smaller pieces that were then sieved to obtain particles between 1 and 2 mm in diameter. The jaw crusher was rinsed with 70% ethanol prior to crushing in the UV-treated laminar flow hood. Particles were loaded into 304 stainless steel cylinders (25.4 cm length, 1.27 cm diameter, 800 µm mesh size (56)), rinsed with MQ water, wrapped in foil, and dry autoclave-sterilized. The coupon containing rhyolite chips was deployed to a depth of ∼100 m in Grant B944 on 2021 May 21 (Julian date 147) using 1.2 mm diameter vinyl coated stainless steel wire. During sampling on 23 June (174), the coupon was brought to the surface and stored in anoxic distilled water in a rubber stopper capped polyvinyl chloride (PVC) tube. After sampling on that day and while still in the field, the coupon was redeployed to the same depth. The coupon was recovered on 2021 August 25 (237) and immediately transferred in the field to an ethanol (70%)-treated and MQ water-washed PVC tube, capped with PVC caps, and frozen on site using dry ice during transport back to the lab. At the lab, tubes and their contents were kept at −80 °C until processing.

The coupon and its contents were thawed at room temperature, opened, and rhyolite particles were removed using flame-sterilized forceps. Particles (∼500 mg total) were subjected to DNA extraction and quantification as described above. PCR amplification of bacterial and archaeal 16S rRNA gene fragments utilized the universal 515F (5′ GTGYCAGCMGCCGCGGTAA 3′) and 806R (5′ GGACTACNVGGGTWTCTAAT 3′) primers and cycling conditions as previously described (57). Sequences were assembled and subjected to quality filtering, and removal of singleton sequences, as previously described (57), resulting in 583,592 reads (Table S6). Reads were clustered into OTUs using a 97.0% similarity cutoff, and OTUs were classified using Silva 138 SSU database.

### Seismic energy

The original goal of this project was to obtain energetic data measured using down-borehole instrumentation installed in B944 for downstream analyses. However, B944 lost communication/data during the 2021 sampling season. As such, all seismic data for the YVFP region was obtained from the Quarterly Seismicity Summaries released publicly by the University of Utah (https://quake.utah.edu/earthquake-center/quarterly-seismicity-reports). Location (global position coordinates [GPS]), the computed local Richter magnitude (M), and depth below sea level (km) of each seismic event were compiled. Location and depth data for each event epicenter were used to quantify the distance to B944. Specifically, the surface distance between the epicenter and borehole locations was calculated using GPS coordinates and assumed a Vincenty ellipsoid surface between the two points. Distance at depth was then calculated from the surface distance and depth measurements using the Pythagorean theorem, considering the depth of sampling at B944. M readings were then converted to energy (J) according to the Richter equation, log_10_J = 4.4 + 1.5M. Energy was then normalized to distance using the inverse-square law (1/*D*^2), where *D* is the distance in km. The date, location, *M*, and energy of seismic events are reported in Table S7.

### Precipitation data

The original goal of this project was to obtain precipitation data measured with an onsite weather station installed at B944 for downstream analyses. However, B944 lost communication/data during the 2021 sampling season. As such, precipitation data was compiled from the Cabin Creek, Yellowstone National Park, Wyoming (44.3113889; −110.15027778; Elevation, 2646 m) weather station administered by the Climate Center, Desert Research Institute, Reno, Nevada (NESS ID = FA45D070; National Weather Service identifier = 480118; https://raws.dri.edu/cgi-bin/rawMAIN.pl?wyWCAB). The Cabin Creek weather station is located 32.5 km from the Grant B944 borehole and is on the southeastern part of the lake. Precipitation data is reported in Table S8.

### Statistical tests

Multivariate approaches were used to examine relationships between seismic energy absorbed at B944, geochemistry, and community composition. The seismic energy absorbed (in kJ) was summed between sampling dates. In the case of the first sampling date, the average time between the second through fifth sampling dates (40 days) was used to identify the window of time preceding the first date of sampling (2021 April 17) to quantify energy released over that interval (SI Appendix Dataset S7). The same approach was taken for precipitation data (SI Appendix Dataset S8). The summed energy (kJ) or precipitation (mm) preceding the first sampling and between the next four samplings were then used to create Euclidean dissimilarity matrices for each dataset. Geochemical analytes that did not vary among sampling dates were not considered in statistical tests, nor were analytes that were only detected on a single sample date. To limit redundancy, only unfiltered sulfide and unfiltered ferrous iron data were used in statistical tests. Isotopic and concentration data for DOC were excluded from analysis since they were only measured on three of the five sampling dates. Finally, isotopic analyses of H_2_O were excluded from analyses as these are unlikely to influence microorganisms. The remaining geochemical data (36 analytes, Table S9) were then scaled (normalized) to values of 0 to 1 by dividing by the maximum measured value, and the resulting dataset was used to generate a Euclidean dissimilarity matrix. MAG OTUs that were detected in communities from only one sampling date were excluded and the relative abundance of the remaining OTUs (91–99% of binned populations) were used to generate a Bray-Curtis dissimilarity matrix. Mantel regressions were performed to determine correlations between matrices using PAST v4.16c.

### Mechanical comminution of rhyolite

Roughly 1 inch cube subsamples of unaltered and altered rhyolite (described above) were cut using a Mk-14 diamond blade water saw (MK Diamond Products, Inc., Torrence, CA). The surface of each cube was washed with 70% peroxide to remove organic contamination. Cubes were then ground to 0.5 to 2 mm particles using an ethanol-treated jaw crusher (described above) in an ethanal- and UV-treated laminar flow hood. In triplicate, roughly 40 g of the coarse material was then transferred to a prechilled (4 °C) and ethanol (70%)-rinsed Pulverisette 6 mill (Fritsch, Pillsboro, NC). The chromium hardened stainless steel bowl and its contents were flushed with filter-sterilized (0.2 µm nylon button filter) UHP N_2_, prechilled to 4 °C, and then was subjected to milling for 5 min at 500 rpm to generate a fine powder. A 10 mL subsample of the headspace from the mill was collected and analyzed via GC as described above to quantify gases released from inclusions or pore spaces (dry crush). Subsamples of the <63 µm fraction were subjected to X-ray fluorescence (XRF) and X-ray diffraction (XRD) analysis to semi-quantitatively estimate elemental and mineralogical compositions of altered and unaltered rhyolite. XRF was conducted using a portable Niton XL3t 950 unit (ThermoScientific, Waltham, MA) and XRD was conducted using a SCINTAG X-1 system (Eigenmann GmbH, Mannheim, Germany). Importantly, elements lighter than 24 amu cannot be detected using this portable XRF unit due to interference by shielding.

Generation of H_2_ from pulverized rock was determined in the presence of water (wet crush), using previously described methods (10). Briefly, 10 g of fine powdered rock (<63 µm) was distributed into serum bottles containing 20 mL of anoxic, MQ water, leaving a headspace of 13 mL. The headspace of vials was then purged with UHP N_2_. In the first set of experiments, serum bottles were incubated at 35 °C (temperature of sampled B944 fluids), and subsamples of headspace were collected following 24, 48, and 120 h incubation. Subsamples of gas were collected by first injecting 10 mL of UHP N_2_ into the headspace, allowing it to mix for 5 min, and then removing 10 mL of gas to run on the GC. After 120 h incubation, the liquid phase of reactors was collected via filtration (0.2 µm Supor filters) and was preserved for determination of DOC concentration and its isotopic (δ^13^C) composition, as described above. All gas or aqueous concentration data were normalized to the amount of milled rock added to each vial. The pH of the remaining water, following collection of subsamples for DOC, was measured with pH strips.

## Results and discussion

### Overview of sample collection

From 2021 May 27 to November 3 (Julian days 147 to 307), five fluid samples were collected from a depth of ∼100 m (325 ft) in B944. Fluids were collected using a bladder pump via displacement with compressed air and subsamples were preserved for geochemical characterization, planktonic cell quantification, and metagenomic sequencing of planktonic cells. Earthquake catalog data were compiled from the Quarterly Seismicity Summaries released publicly by the University of Utah and were used to calculate the kinetic energy absorbed at the borehole and the energy for each seismic event was summed over six sampling windows that predate the first sample collection and that then span each additional sample collection date, as reported in Table S7. Similarly, precipitation data were compiled from the Cabin Creek weather station near the outlet of Yellowstone Lake and precipitation amounts were summed over six sampling windows that predate the first sample collection and that then span each sample collection, as reported in Table S8. Statistical analyses were used to identify associations among the datasets.

#### Influence of seismicity and precipitation on B944 fluid geochemistry

**Fig. 2.**
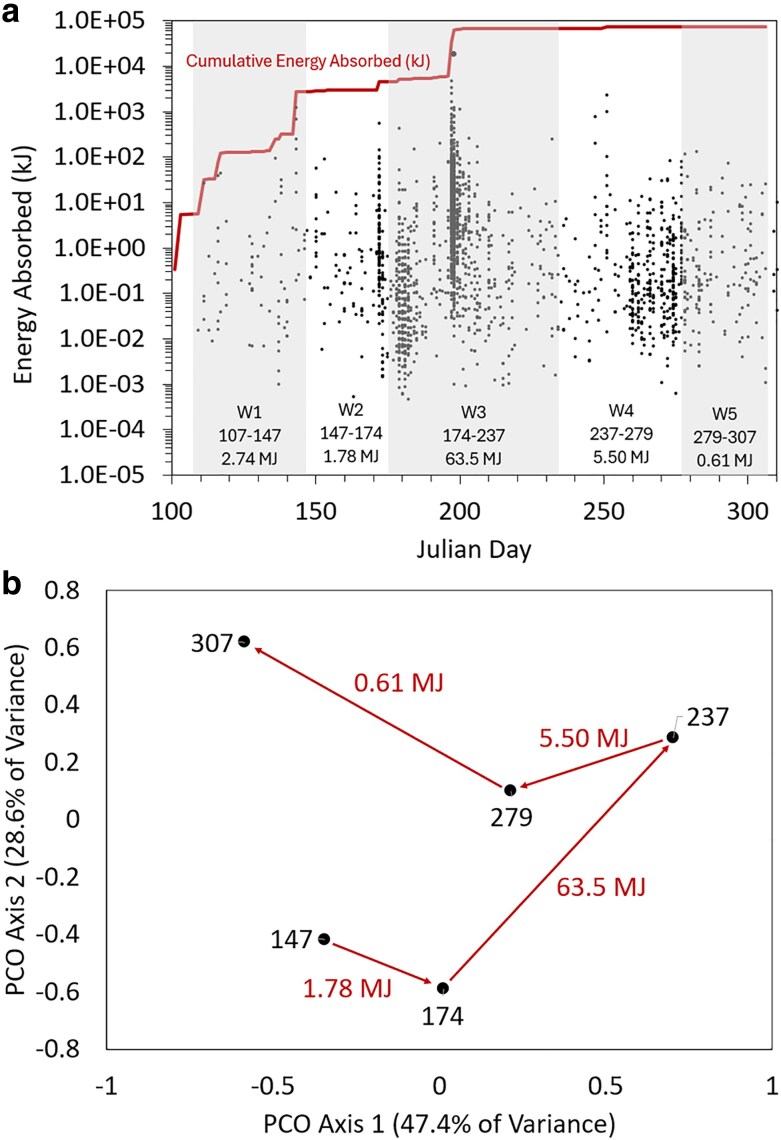
Kinetic energy absorbed at the Grant B944 (a) borehole during the defined sampling windows (W) in 2021. The cumulative seismic energy was tabulated for 10 days prior to the start of the first sampling window and is indicated for each of the five sampling windows that precede each sampling collection. Seismic and estimated kinetic energy data are reported in Table S7. Principal coordinate analysis (PCO) of the variation in gas and aqueous geochemical data for B944 (b). The Julian date for each sample is indicated and arrows indicate the progression of geochemical change associated with the cumulative kinetic energy absorbed during the specified sampling window. Geochemical data are reported in Table S1 and normalized geochemical data used to construct PCO plots are reported in Table S9.

A total of 2182 seismic events with an average magnitude (M) of 0.9 and a maximum M of 3.6 were detected between 2021 May 13 and November 3 (Julian days 133 to 307) at B944 (Table S7). These events combined for a calculated total energy absorbed of ∼74.2 megajoules (MJ) over this time interval. The highest frequency (1416 events) and energetic intensity (M 3.6) of events occurred between days 174 and 237 (third sampling window), totaling ∼64 MJ absorbed (Fig. 2a); the majority of which were associated with a swarm on days 196 to 197 (369 events, 29.4 MJ). Another swarm occurred just prior to the third sampling window on days 172 to 173 (139 events, 1.5 MJ). Many of these events were concentrated near the southern caldera boundary in Yellowstone Lake (Fig. 1a). Precipitation data was also compiled over the sampling windows, which totaled 207.74 mm at the Cabin Creek weather station located 32.5 km southeast of B944 (Table S8). Most of the precipitation occurred in the 40-day window (68.29 mm total) preceding the first sampling (Julian day 147) and during the 63-day window (76.31 mm total) preceding the third sampling (Julian day 237). Sampling across these seismic and precipitation events was fortuitous and provided the unique opportunity to evaluate whether coordinated geochemical and microbial changes in aquifer fluids were associated with these seismic and precipitation events.

Concentrations of SO_4_^2−^ and Cl^−^, often used to identify a hydrothermal influence on natural waters (Fig. S1) (58), in B944 waters were low (63 and 310 µM or 6 and 11 mg L^−1^, respectively, Table 1) and were similar to concentrations measured when the borehole was originally drilled in 2008 (43). Likewise, the conductivity (230 to 356 µS cm^−1^) of waters across the sampling interval was similar to when the borehole was originally drilled (267 µS cm^−1^), but low compared to typical hydrothermal aquifer waters in Yellowstone (>1 mS cm^−1^), as estimated via discharge in surficial hot springs (58). However, the δ^18^O and δ^2^H in H_2_O of waters were isotopically lighter than when the well was originally drilled but were similar to nearby Yellowstone Lake (Fig. S2). Finally, the temperature (∼35 °C) at depth and pH (∼7.5 at the top of the water column in the well) of waters were like those (31 to 35 °C; pH 7 to 9) when the well was originally drilled. Together, these data point to aquifer waters intersected by B944 as being meteoric (58) and heated by a shallow source like other aquifers in the Grant area of the YPVF (59).

A total of 36 geochemical analytes that varied among sampling dates and that were above limits of detection in more than two of the samples were used to generate a Euclidean matrix describing the dissimilarity in geochemistry among fluid samples (Table S3). Over the sampling period, changes in the bulk geochemical composition of fluids were observed in relation to seismic energy but not in relation to precipitation data. A Mantel regression of matrices describing the Euclidean dissimilarity in accumulated seismic energy absorbed and geochemistry revealed a significant, positive relationship (Mantel *R* = 0.55, *P* = 0.02). However, a mantel regression of matrices describing the Euclidean dissimilarity in accumulated precipitation and geochemistry revealed an insignificant, inverse relationship (Mantel *R* = −0.24, *P* = 0.78). Principal coordinates (PCO) analysis of the Euclidean matrix describing the dissimilarity in geochemistry also revealed systematic changes in geochemical composition (Fig. 2b). PCO axis 1, which explained 47.4% of the variance, was significantly correlated with seismic energy absorbed over those sampling windows (adj *R* = 0.82, adj *P* = 0.02; Fig. S3a) and this relationship was driven largely by changes that occurred between days 174 and 237 (Table 1). Neither axes 1 nor 2 of the seismic energy PCO plot were significantly associated with accumulated precipitation.

Among those geochemical analytes that varied most and that could provide insight into microbial community changes due to their use in or generation by microbial energy metabolism were H_2_, sulfide, and dissolved organic carbon (DOC). Concentrations of these analytes increased between day 147 and day 237, then decreased thereafter to day 307. Over this time frame, H_2_ concentrations ranged from 27 to 44 µM, roughly 10 to 20 times the highest concentration measured in YPVF waters (thermal and nonthermal) to date (47). Increases in H_2_ were accompanied by increases in sulfide, possibly suggestive of microbial coupling of H_2_ oxidation to SO_4_^2−^ reduction, as discussed below. In addition to sulfide concentration increasing over the sampling period (except for the last sample), the fraction of total sulfide (nonfiltered) as soluble sulfide (0.22 µm-filtered) systematically increased from 30.1 to 79.6% of total during the sampling period, possibly indicative of its recent input into the system. The DOC concentration increased and the δ^13^C-DOC systematically decreased (−14 to −18‰ vs. PDB) across the sampling period (Table 1; Fig. S4b), indicating dilution of the existing pool with isotopically light DOC. DOC concentrations in B944 waters (3.5 to 7.9 mg L^−1^) were substantially higher than in thermal and nonthermal waters in the YPVF with similar pH (Fig. S4a; Table S10) and the δ^13^C-DOC was substantially heavier than most hot springs sourced by meteoric waters (i.e. −18 to −28 ‰ vs. VPDB) (60). It is possible that the increase in DOC concentration can be attributed to biotic fixation pathways, as potentially indicated by a decrease in the δ^13^C-DOC, if DIC was also isotopically heavy. However, DIC concentration (and its δ^13^C) was not measured. Alternatively, it is possible that this observation is due to dilution of the existing DOC pool by input of isotopically light (abiotic or biotic) DOC due to alteration of fluid flow paths or release from fluid inclusions. Both possibilities are discussed in more detail below.

While it has been shown that the conductivity of deep waters (>200 m) impacted by hydrothermal activity can shift seasonally (61), this is not likely to be responsible for the geochemical changes observed in B944 herein, at least on the time frame of the measurements made. For example, if seasonal recharge was responsible for the observed geochemical changes, the changes would be expected to be reflected in systematic shifts in the isotopic composition of water, concentrations of conservatively behaving dissolved ions (i.e. Na^+^, Cl^−^, K^+^), and electrical conductivity, none of which were observed (Table 1; Fig. S2). Further, and as described above, the observed systematic geochemical changes were not associated with accumulated precipitation during the time windows preceding sampling events. While seismic energy may induce near instantaneous changes to aquifer hydrology, such as has been shown by discharging hot springs sourced by shallow and deep aquifers in the YPVF (62, 63), far less is known of the timescales of recharge of Yellowstone's subsurface aquifers. A recent isotopic analysis of an acidic hot spring in Yellowstone and a colocalized circumneutral hot spring sourced predominated by a near surface aquifer and a deep aquifer, respectively, revealed timescales of aquifer recharge that was on the order of years (acidic spring) to hundreds of years (circumneutral spring) (64). Likewise, several hot springs in the Norris Geyser Basin of Yellowstone that are at least partially sourced by near surface aquifers exhibited temporal geochemical changes that were associated with seasonal precipitation events (57) or changes in the water table (65). While the hydrological flow paths of aquifers intersected by B944 are not known, the near surface water bearing units near B944, as inferred from aerial electromagnetic data (Fig. 1b) (40), likely can facilitate recharge and lateral fluid flow. However, it is possible that the time intervals associated with recharge and flow are longer than those observed in other areas of YNP and those were not captured in the time frame of the temporal study conducted herein. These observations call for additional investigations to better constrain the hydrological dynamics of thermal and non-thermal aquifers, in particular considering predicted future changes in precipitation across the YPVC (66).

#### Influence of seismicity and geochemistry on microbiology

**Fig. 3.**
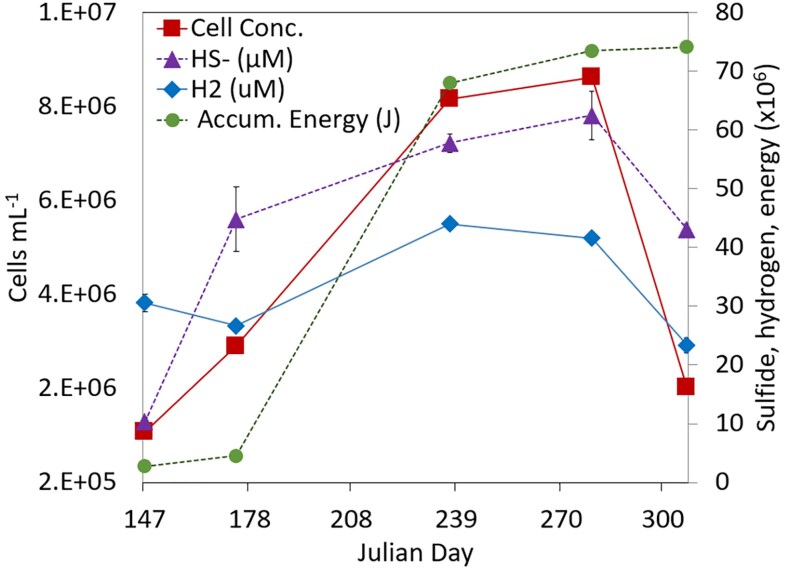
Concentrations of sulfide (HS^−^), hydrogen (H_2_), and cells in samples collected on specified dates and the accumulated energy absorbed as calculated from seismic data. The reported HS^−^ values are on unfiltered samples. Chemical and microbial data depict the average and standard deviation of three triplicate measurements for each analyte (see Tables 1 and S1). Accumulated energy (same as presented in Fig. 2) is also included to facilitate alignment of seismic energy absorbed at B944 and geochemical and microbial changes in B944 fluids.

Shifts in the geochemical composition of waters were accompanied by increases in the concentration of planktonic cells (Table 1; Fig. 3). Cell concentrations increased systematically from 1.28 × 10^6^ cells mL^−1^ on day 147 to 8.36 × 10^6^ cells mL^−1^ on day 237, stabilizing thereafter and then declining to 2.23 × 10^6^ cells mL^−1^ on day 307 following the near cessation of seismic activity (Fig. 2a) and a shift in geochemistry toward its initial composition (Fig. 2b). Filtered biomass from each sampling date was subjected to DNA extraction, metagenomic sequencing, assembly, and binning into MAGs (Table S2). MAGs were then organized into OTUs based on 95% ANI and their relative abundances were determined by read mapping (Table S4). MAG OTUs were characterized taxonomically (Table S4) and this yielded similar results to assembly-free taxonomic analyses (Fig. S5).

B944 communities had similar genomic richness across sampling dates, ranging from 66 to 75 OTUs. Only 38 of those OTUs, however, were identified in communities from all five sampling dates, indicating high population turnover between sampling windows. This is interesting as several recent studies suggest relatively stable subsurface communities hosted in continental bedrock (61) but that can drastically change when physical changes in the permeable fracture network occur (67). A dissimilarity matrix was constructed to describe variation in the distribution and abundance of OTUs across sampling dates (Table S9). Regression of this matrix and a matrix describing variation in the geochemical composition of fluids revealed a positive (albeit statistically insignificant) correlation (Mantel *R* = 0.49, *P* = 0.13). Given that the geochemical matrix is also correlated with the seismic energy matrix, this suggests that seismic activity is influencing the microbial community composition through its effect on aquifer geochemistry. Alternatively, it is possible that changes in fluid flow paths due to seismic energy allowed for planktonic cells of slightly different abundance and composition to migrate into and out of the B944 fracture waters.

**Fig. 4.**
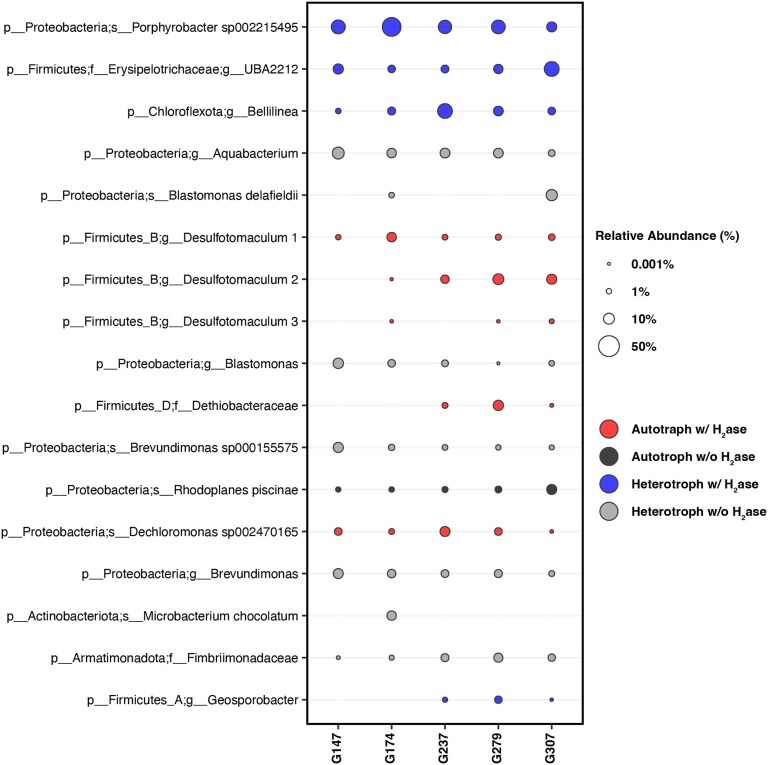
Relative abundance of metagenome assembled genome (MAG) operational taxonomic units (OTUs) in planktonic communities sampled from the B944 borehole in 2021 as a function of sampling date (Julian date). Bubbles are overlaid based on MAG OTUs encoding autotrophic pathways and/or homologs of hydrogenase enzymes predicted to be involved in H_2_ oxidation (see Table S5).

To determine if shifts in community composition were related to measured geochemical changes, the taxonomic identities and metabolic potentials of representative MAGs for abundant MAG OTUs were characterized (Fig. 4). At a taxonomic level, the dominant populations were closely related to organisms common to aquatic and/or subsurface environments, including heterotrophic OTUs related to *Porphyrobacter*, *Bellilinea*, *Brevundimonas*, and *Aquabacterium* and facultative autotrophic OTUs related to *Dethiobacteraceae* (encodes the Wood-Ljungdahl (WL) pathway), *Dechloromonas* (Calvin cycle), and *Rhodoplanes* (Calvin cycle) (Table S5). *Desulfotomaculum* (3 OTUs), like several other characterized members of this genus (68), encodes a partial WL pathway (methyl branch) and can likely fix CO_2_ if an organic carbon source such as acetate is available (i.e. mixotrophic). The abundances of these OTUs shifted markedly over the sampling period, most notably via a decrease in *Porphyrobacter* from 39 to 17% of the binned reads between days 174 and 237 and an increase in *Bellilinea* from 4 to 22% of binned reads over this same time. Similarly, the abundance of the putatively mixotrophic, bisulfite reductase-encoding sulfate reducer *Desulfatomaculum* population 1 decreased from 7 to 1%, whereas *Desulfatomaculum* population 2 increased in abundance from 0 to 5% of the binned reads over this same time; the third *Desulfatomaculum* population was variably present in low abundances over the sampling period. Consistent with these metabolic predictions, SO_4_^2−^-reducing enrichments using waters from B944 that were amended with H_2_/CO_2_ or a mixture of formate, lactate, acetate, and propionate both produced a culture of *Desulfatomaculum* (69), that was most closely related (96% sequence identities; NCBI Accession: OP895697) to the 16S rRNA gene from the population 2 MAG. The *Dethiobacteraceae* MAG, which encoded homologs of proteins involved in thiosulfate reduction (PhsA; Table S5), increased in abundance between days 237 and 279, decreasing thereafter.

When summed across OTUs, the abundance of putative autotrophs decreased initially from 13% of the binned community on day 147 to 8% on day 174, then increasing to 17 and 24% on days 237 and 279, respectively, when most of the seismic energy was absorbed (Fig. 2a), followed by a decrease on day 307 (Fig. S6) when seismic activity dissipated. Similarly, the abundance of autotrophic MAGs that encode [NiFe]- and [FeFe]-hydrogenases increased from 12% of the community on day 147 to 23% on day 279, decreasing to 14% on day 307. These increases were largely driven by increased abundances of autotrophic/mixotrophic MAG OTUs related to *Desulfatomaculum* (all three populations encode six homologs of [FeFe]-hydrogenase and one homolog of [NiFe]-hydrogenase) and *Dethiobacteraceae* (encodes four homologs of [FeFe]-hydrogenase and one homolog of [NiFe]-hydrogenase). The number of hydrogenase homologs encoded in these two MAGs points to their dependence on H_2_, like that of the common subsurface facultatively autotrophic sulfate reducer *Candidatus* Desulforudis audaxviator (encodes six homologs of [FeFe]-hydrogenase and two homologs of [NiFe]-hydrogenase) (70, 71). Given that hydrogenases are inherently reversible (54), it is also possible that these taxa contribute H_2_ to the system when electron acceptor availability is low. Collectively, these observations suggest that the increase in the concentration of cells was driven, at least in part, by increased H_2_-driven chemolithotrophic activity, as evidenced by increases in the relative abundance of putative H_2_-dependent autotrophs and DOC and a decrease in the ^13^C of that DOC. The increase in sulfide and H_2_ over this time, in particular between days 174 and 237, suggests this increase in cell concentration may have been stimulated by chemolithotrophs supported by the H_2_/SO_4_^2−^ or H_2_/S_2_O_3_^2−^ redox couples. Alternatively, if DOC is exogenously being introduced to the aquifer intersected by B944 via changes in fluid flow paths, it is possible that these organisms switch to heterotrophic metabolisms and may be responsible for production of H_2_.

### Rhyolite as a colonizable substrate

To evaluate the ability of microorganisms to colonize relevant bedrock (i.e. rhyolite, the predominant bedrock intersected by B944 (43)), unaltered rhyolite chips (0.5–2 mm diameter) retained in a stainless-steel coupon were incubated for 90 days at a depth of ∼100 m in B944. The incubation period spanned the first sampling date (day 147) to the third sampling date (day 237). Unaltered rhyolite was subjected to XRF and XRD analyses to semiquantitatively determine elemental and mineralogical composition, and these data are reported in Tables S11 and S12, respectively. Following incubation, genomic DNA was extracted from the rock chips and subjected to PCR amplification and sequencing of 16S rRNA genes to determine if the taxonomic composition of communities that colonized rhyolite surfaces resembled those of the planktonic communities analyzed above using metagenomic approaches.

The dominant 16S rRNA gene OTU (53% of 16S rRNA gene sequences) identified in the rhyolite-associated community was related to *Dethiobacteraceae* (Table S6). While the *Dethiobacteraceae* MAGs that form the OTU from B944 do not include 16S rRNA genes, 16S rRNA gene fragments in unbinned contigs with similar coverages to that of the MAG OTU share 97–99% identities with those recovered from the rhyolite chips. The maximum abundance of the *Dethiobacteraceae* OTU MAG was 8.6% in planktonic communities (Table S4), suggesting that this organism may prefer growth on surfaces such as rhyolite. Additional abundant OTUs (>2% of total reads) associated with the rhyolite chips were those related to *Brevundimonas* (8%), *Geosporobacter* (7%), *Desulfotomaculum* (6%), *Rhodocyclaceae* (5%), and *Bellilinea* (3%), all at abundances like those observed for MAG OTUs from filtered waters collected across sampling dates. While *Porphyrobacter*, *Bellilinea*, and *Aquibacterium* MAG OTUs were abundant among planktonic communities across all sampling dates (17.0 to 39.0%, 1.1 to 21.7%, and 2.5 to 12.7%, respectively), 16S rRNA gene OTUs affiliated with these genera were not abundant (<4%) in rhyolite-associated communities. This may suggest these populations prefer growth in a planktonic state. It is also possible that the differences in the abundances of OTUs in planktonic communities assessed via metagenomics and rhyolite surface-associated communities assessed via 16S rRNA amplicon sequencing could be attributable to PCR primer amplification bias. Nonetheless, overall similarities in the composition of the communities suggest that planktonic communities sampled over the course of the study represent, at least in part, those that may associate with mineral surfaces in the subsurface fracture networks that form the near surface aquifer that is intersected by B944.

### Rhyolite as a source of H_2_ and DOC

**Fig. 5.**
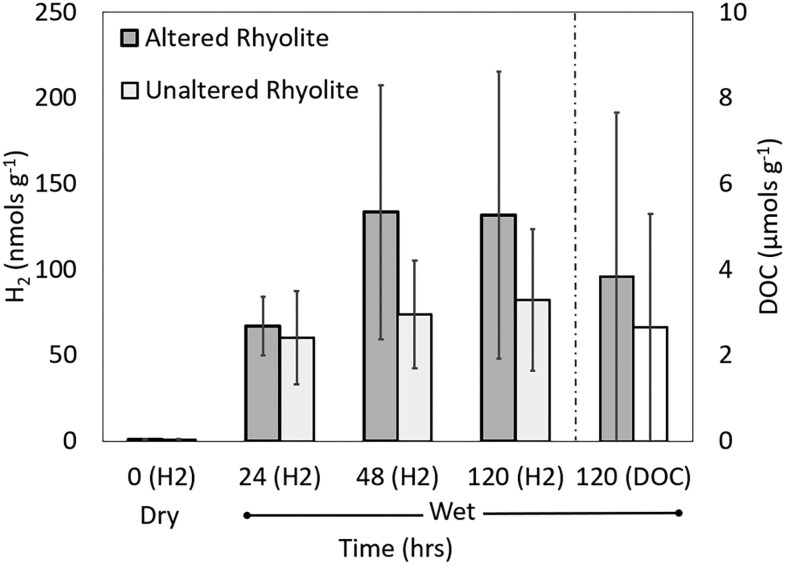
Concentrations of hydrogen (H_2_) and dissolved organic carbon (DOC) in altered and unaltered Lava Creek Tuff (rhyolite) hand samples. Hand samples were pulverized dry and the concentration of H_2_ was measured (0 h, dry). Then, pulverized rock was incubated in water at 35 °C (wet) over a period of 120 h with subsamples of gas sampled for H_2_. At the end of the incubation, the aqueous phase was analyzed for DOC, as indicated by the hashed line.

To determine the potential for release of organic carbon and H_2_ from rhyolite and to examine whether H_2_ can be generated via mechanoradical silicate-based mechanisms (i.e. cataclasis (10–12)), both unaltered and altered Lava Creek Tuff (rhyolite) were subjected to pulverization and exposure to water followed by analysis of the gas and liquid phase. The elemental and mineralogical compositions of the starting materials are given in Tables S11 and S12. While the elemental compositions were highly similar, altered rhyolite had a higher amount of opal/silica hydrate and kaolinite than the unaltered rhyolite, likely driven by acid-steam hydrothermal alteration (72, 73). A previously described approach was used, wherein coarsely ground rock was pulverized to a grain size below 63 µm in a sealed ball mill at 4 °C in the presence of UHP N_2_, with the gases released from this dry crush considered to be released from mineral pore spaces, fluid inclusions, and/or crystal structures (10, 24). A total of 0.41 ± 0.71 and 0.37 ± 0.65 nmols H_2_ g^−1^ was quantified following dry crushing of altered and unaltered rhyolite, respectively (i.e. the 0-day time point; Fig. 5). These values are low when compared to those measured from silica-rich quartz and quartzite (∼10 nmols g^−1^) when pulverized to a similar grain size (24). However, those H_2_ amounts reported previously were exposed to water followed by immediate subsampling whereas those reported here were not exposed to water. It is possible that, in the previous studies, exposure to water promoted release of H_2_ from mineral surfaces due to disruption of van der Waal interactions (74).

Following dry crushing, pulverized rock powder (10 g) was transferred to reactors containing water (20 mL) that were incubated at 35 °C. While H_2_ production was highly variable among replicates for each rock type, similar to what has been observed previously with rock hand samples when pulverized and exposed to water (10), the average H_2_ concentration among the three replicates increased linearly up to 48 h incubation for both rock types and then plateaued. The average rate of H_2_ production over this period was 2.77 and 1.54 nmol g^−1^ h^−1^ for altered and unaltered rhyolite, respectively (Fig. 5). These rates are roughly five and three times faster than those reported for pulverized quartzite (0.42 nmol g^−1^ h^−1^) when incubated at 35 °C (10). The water/rock ratio in experiments conducted here (2:1) was double that used in the previously mentioned study (1:1), which may suggest that availability of water limited rates of H_2_ generation in the former study. Small quantities of CH_4_ and CO_2_ were also released from the rocks but were near or below values from the calibration standards and thus are not reported.

At the end of the 120 h incubation of pulverized rock powder in the presence of water, the aqueous phase was harvested and the pH of water in the reactor, DOC concentration, and the δ^13^C of DOC were determined. The pH of the water in reactors containing altered rhyolite, as determined semiquantitatively with pH paper, was ∼5.5 and that of unaltered rhyolite was ∼6.5. While highly variable, the average DOC concentration in altered and unaltered rhyolite following pulverization and incubation in the presence of water was 3.8 ± 3.5 and 2.7 ± 2.5 µmol g^−1^. Due to the experimental design, it is not known whether the DOC was released from pore spaces, fluid inclusions, and/or crystal structures or whether it was generated during the incubation via water/rock/gas reactions. However, during the dry pulverization of the mineral exceedingly small amounts of CO_2_ (only detectable in one of the three altered rhyolite replicates but below calibration standards) were released into the UHP N_2_ atmosphere of the reactors. Thus, it is likely that the DOC that was detected was present in pore spaces, fluid inclusions, and/or crystal structures was released during pulverization. While the DOC concentration was highly variable, the δ^13^C of DOC was less variable and averaged −14.7 ± 1.3 and −13.6 ± 0.9 ‰ vs. VPDB for altered and unaltered rhyolite, respectively. Interestingly, these values are within the range of being attributable to biological activity if the δ^13^C of DIC that was fixed was similar to what is typically encountered in YPVF (75). However, hydrothermal synthesis reactions have been shown to produce DOC that has similar isotopic composition (e.g. (76)), rendering it difficult to attribute the detected DOC to a biotic or an abiotic source. Further, microorganisms have been shown to inhabit the pore spaces and microfractures of highly crystalline rock (e.g. (77, 78)) suggesting that the DOC released from porous rhyolite could be from endolithic cells lysed during pulverization. While it is tempting to speculate that DOC released from rhyolite due to seismic induced fracturing stimulated the increase in the concentration of microbial cells, additional studies are needed to determine the composition and lability of the DOC and whether it represents a bioavailable carbon and energy source for those cells.

## Conclusions

Observations made here suggest that kinetic energy radiated from local earthquakes altered the geochemical and microbial compositions of aquifer fluids. Fortuitous sampling in the seismically active YPVF allowed for the apparent capture of the chemical and microbial response to the onset of a seismic swarm in 2021 and its conclusion, with the fluid chemistry and microbiology beginning to return to their initial compositions after the final ∼28 days of relative quiescence in seismicity. While it is not known if the change in fluid composition was due to alteration of fluid flow paths, mineral resurfacing and subsequent water–rock interaction, or release from fluid inclusions or pore spaces in rock, the relatively short timescales over which the changes occurred may point to flow path alteration as a likely explanation, such as has been suggested for surface hydrothermal features in Yellowstone (62, 63). Resistivity mapping of the near subsurface via aerial electromagnetic surveys near the Grant B944 borehole reveal plausible lateral fluid conduits that could enable fluid migration (40). While fluid flow path modification would likely be permanent, the observation that the chemical composition of fluids began to return to near starting conditions following quiescence in seismicity may alternatively point to an important role for seismic-induced cataclasis in promoting increased water-rock interaction and fluid inclusion release that temporarily modified the chemistry of the fluids. Additional higher frequency sampling, alongside more detailed subsurface geophysical measurements, would provide useful insight into evaluating each of these possibilities.

Changes in the geochemical composition of fluids were coordinated with changes in the taxonomic composition of communities and their functions, particularly those putatively involved in dark (chemolithotrophic) primary production. Putative SO_4_^2−^/S_2_O_3_^2−^-reducing chemolithotrophs related to the *Dethiobacteraceae* family and *Desulfatomaculum* genus, both of which encode multiple hydrogenase homologs and that encode CO_2_ fixation pathways, increased in abundance over time. The systematic increase in the abundance of putative hydrogenotrophic chemolithotrophs, combined with the increase in DOC concentrations and concomitant depletion of δ^13^C of that DOC, suggests that the activity of these taxa was stimulated, perhaps by bedrock fracturing that promoted generation of H_2_ in aquifer fluids. Rhyolite, the predominant bedrock intersected by B944, was preferentially colonized by *Dethiobacteraceae,* suggesting that its role as a primary producer may be even greater than what would be estimated based on planktonic community data. Rhyolite was also shown to harbor substantial DOC that, when fractured by seismic activity, could be released into surrounding waters. Increased production of DOC by H_2_-dependent chemolithotrophs (e.g. *Dethiobacteraceae/Desulfatomaculum*) or release of DOC from rhyolitic bedrock, one or both of which appear to be associated with seismic energy, may help explain the abundance of putative heterotrophs in the aquifer intersected by B944. Indeed, the concentrations of H_2_ and DOC were among the highest ever measured in waters in the YPVF, pointing to the continental subsurface as a potentially sizable reservoir of these compounds that, when made available by seismic activity, can sustain subsurface microbial communities in geologically active regions globally. These observations may be extended to other rocky planets where seismic activity has been detected, such as Mars (79), suggesting that the such activity could expand planetary habitability.

## Supplementary Material

pgaf344_Supplementary_Data

## Data Availability

All data are included in the manuscript and/or supporting information.
