# Supplementary material for: Seismic shifts in the geochemical and microbial composition of a Yellowstone aquifer
Source: PNAS Nexus. 2025 Nov 25;4(11):pgaf344. doi: 10.1093/pnasnexus/pgaf344 (PMC12645456; doi:10.1093/pnasnexus/pgaf344)
Supplement: pgaf344_Supplementary_Data [file pgaf344_supplementary_data.zip › PNASNEXUS-PNASNEXUS-2025-00918-TR-s01.pdf]

SUPPLEMENTARY FIGURES

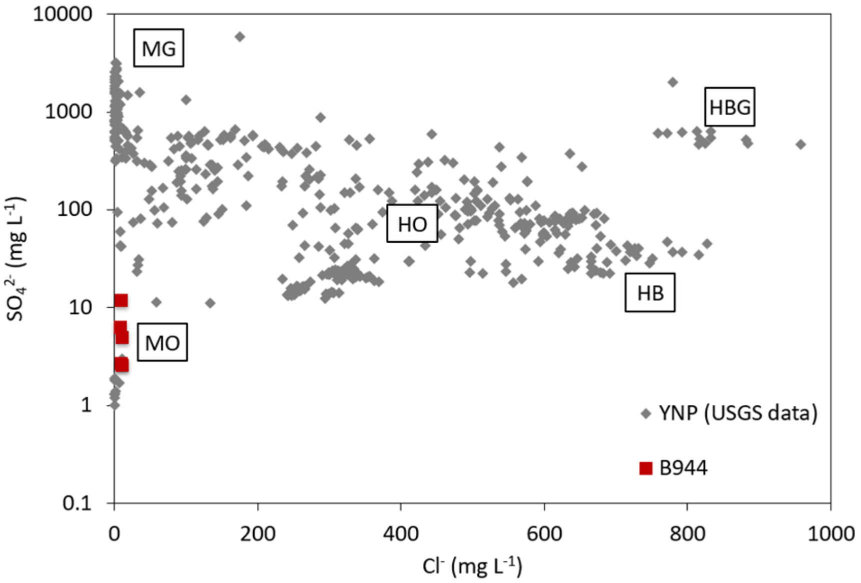

Figure S1. Sulfate (SO_4^{2-}) and chloride (Cl^-) concentrations in sampled borehole waters. B944 waters are plotted as red squares alongside previous data collected from YNP springs between the years 2003-2013 (grey diamonds) are included for reference(1, 2). Abbreviations: MO, meteoric only; MG, meteoric plus hot gas discharge; HO, hydrothermal only (deep hydrothermal reservoir); HB, hydrothermal only with subsurface boiling; HBG, hydrothermal only with subsurface boiling and hot gas discharge.

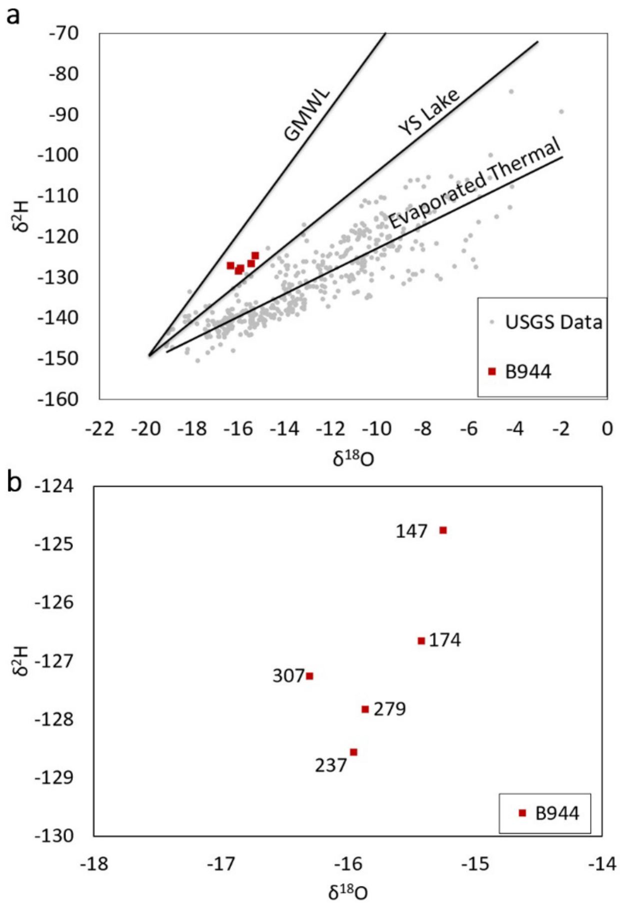

Figure S2. The isotopic composition of hydrogen (δ^2H) plotted relative to the isotopic composition of oxygen ($\delta^{18}O$) in borehole water (H_2O) (**A**). B944 waters are plotted as red squares alongside previous data collected from YNP springs between the years 2003-2013 (grey diamonds) are included for reference(1, 2). The global meteoric water line (GMWL), the slope of Yellowstone (YS) Lake waters, and the slope of fractionated Yellowstone thermal waters (YNP thermal) due to evaporative concentration are plotted for reference(3). Only those values from B944 in 2021 are plotted to show the lack of systematic shifts in the isotopic composition of water (**b**).

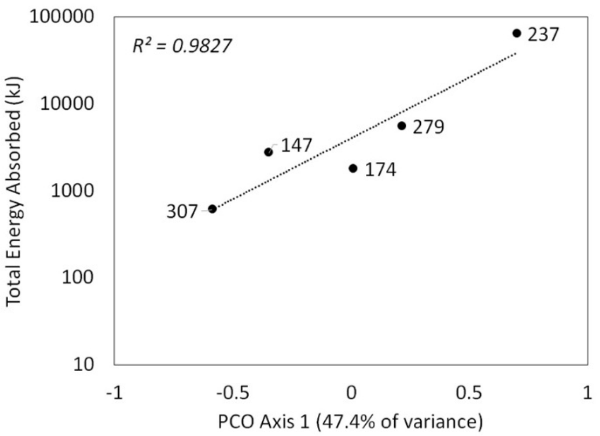

Figure S3. Exponential regression of the total energy absorbed in sampling windows (*see* **Table S7**) as a function of axis 1 coordinates obtained from a principal coordinate (PCO) analysis of a matrix describing geochemical variation in the fluids sampled from the Grant B944 borehole Julian days for each sampling event are indicated. Note that the Y axis is a log scale.

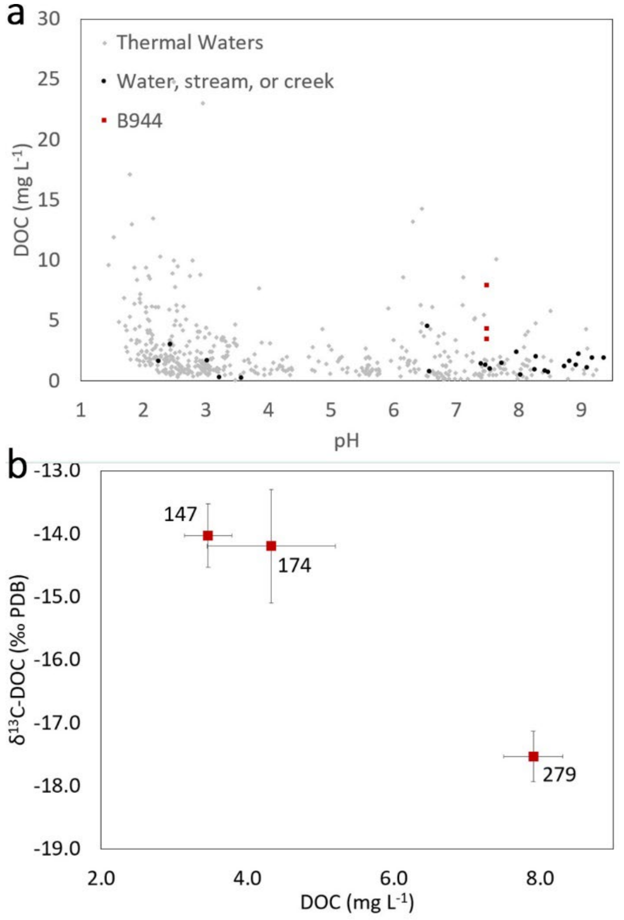

Figure S4. The concentration of dissolved organic carbon (DOC) plotted as a function of the pH of sampled waters (**a**). Select data collected from thermal waters or non-thermal waters (water, stream, or creek) that had a co-registered pH measurement between the years 2003-2013 (grey diamonds) are included for reference(1, 2). These values are reported in **Table S10**. Values for Grant B944 waters are plotted as red squares. The $\delta^{13}C$ of dissolved organic carbon (DOC) plotted as a function of DOC concentration in waters from the B944 borehole with the sampling date indicated (**b**). DOC samples were not collected from B944 on days 237 or 307.

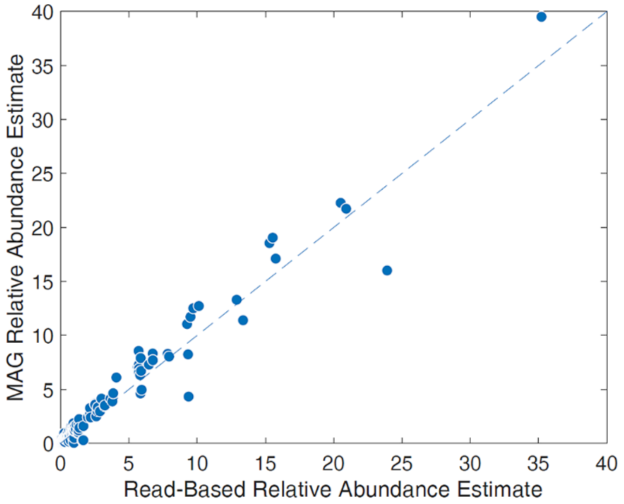

Figure S5. Relationship between metagenome-assembled genome (MAG)-based relative abundance estimates and read-based relative abundance estimates for the Grant B944 communities. 1:1 lines are plotted on each. The R^2 value for a linear regression of the datasets is 0.96 and the p value is $4.1926e^{-206}$. The slope of the relationship is 1.05.

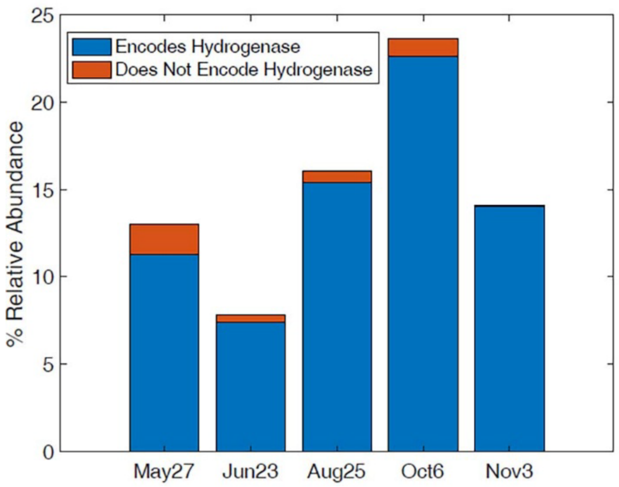

Figure S6. Relative abundance of metagenome assembled genomes (MAGs) that encode autotrophic pathways in communities recovered from the Grant B944 borehole. Bars depicting the abundance of MAGs encoding autotrophic pathways are further demarcated to indicate they encode one or more hydrogenase homologs (blue) or no hydrogenase homologs (orange).

REFERNCES

1. J. W. Ball, R. B. McCleskey, D. K. Nordstrom, J. M. Holloway, 2008, (2006) Water-chemistry data for selected springs, geysers, and streams in Yellowstone National Park, Wyoming, 2003-2005. ed O.-F. R. 2006–1339 (United States Geological Survey), p 137.
2. R. B. McCleskey *et al.* (2014) Water-Chemistry Data for Selected Springs, Geysers, and Streams in Yellowstone National Park, Wyoming, Beginning 2009. (United States Geological Survey, doi: 10.5066/F7M043FS.).
3. D. K. Nordstrom, R. B. McCleskey, J. W. Ball, Sulfur geochemistry of hydrothermal waters in Yellowstone National Park: IV Acid-sulfate waters. *Appl. Geochem.* **24**, 191-207 (2009).
